# Loneliness among adults with visual impairment: prevalence, associated factors, and relationship to life satisfaction

**DOI:** 10.1186/s12955-019-1096-y

**Published:** 2019-02-01

**Authors:** Audun Brunes, Marianne B. Hansen, Trond Heir

**Affiliations:** 1 Section for Trauma, Catastrophes and Forced Migration - Adults and Elderly, Norwegian Centre for Violence and Traumatic Stress Studies, PB 181 Nydalen, Oslo, Norway; 2 Norwegian National Unit for Hearing Impairment and Mental Health, Division of Mental Health and Addiction, Oslo University Hospital, PB 1039 Blindern, Oslo, Norway; 3 Institute of Clinical Medicine, Faculty of Medicine, University of Oslo, PB 1171 Blindern, Oslo, Norway

**Keywords:** Blindness, Life satisfaction, Loneliness, Visual impairment

## Abstract

**Background:**

Little is known about whether and to what extent loneliness impacts the lives of people with visual impairment (VI). Thus, the aim of this study was to examine the prevalence of and factors associated with loneliness in adults with VI, and to examine its association with life satisfaction.

**Methods:**

This cross-sectional interview study included a probability sample of 736 adults (≥18 years old) with VI who were members of the Norwegian Association of the Blind and Partially Sighted. The interviews took place from January to May 2017, collecting information about sociodemographics, VI characteristics, adverse life events, loneliness (Three Item Loneliness Scale), and life satisfaction (Cantril’s Ladder of Life Satisfaction). The prevalence of loneliness was compared to data obtained from the general Norwegian population (*N* = 14,884; mean age 46.4 years; 50.7% females).

**Results:**

The prevalence of moderate and severe loneliness in the VI population was 28.7% (95% CI: 25.4, 32.1) and 19.7% (95% CI: 16.9, 22.8), respectively. The rates were consistently higher across age groups compared to the general population. Loneliness was associated with younger age, blindness, having other impairments, unemployment, and a history of bullying or abuse. In addition, higher scores on loneliness were associated with lower levels of life satisfaction (fully adjusted β = − 0.48, 95% CI: − 0.55, − 0.41).

**Conclusions:**

Loneliness is common in adults with VI. Strategies capable of reducing loneliness could improve life satisfaction among people who are blind or visually impaired.

**Electronic supplementary material:**

The online version of this article (10.1186/s12955-019-1096-y) contains supplementary material, which is available to authorized users.

## Introduction

Visual impairment (VI) represents a substantial and often irreversible loss in visual acuity or visual field [1]. People with VI are usually classified into congenital and acquired vision loss, and into moderate VI, severe VI, and blindness [1, 2]. Loneliness may be of particular concern for people with VI, as vision is a key sensory modality for interpersonal interactions and social communication. People who are blind or partially sighted have fewer opportunities to learn and modify social skills [3]. In addition, individuals with VI are at risk of disability [4, 5], poor health [5, 6], unemployment [4, 5], low financial income [5], and adverse interpersonal events [7, 8], factors which strongly correlate with loneliness [9, 10].

Several studies have been published about the prevalence and risk factors of loneliness in individuals with VI [11–18]. However, most studies have included samples of elderly. Thus, investigations of loneliness in young and middle-aged adults with VI are scarce. In addition, the literature is inconclusive regarding sociodemographics and VI-related characteristics as risk factors for loneliness [11–13, 16], and no studies have considered factors such as past exposure to traumatic events. A better understanding of predictors of loneliness in individuals who are blind or partially sighted is important and could be useful for targeting professional help to those who need it.

Loneliness is a strong predictor of health and quality of life [10, 19–21]. Research of the general adult population have shown that those who were lonely had lower levels of life satisfaction compared with those who were not lonely [21]. However, to the best of our knowledge, no studies have addressed the possible relationship between loneliness and quality of life in populations with VI.

To fil the knowledge gap, we conducted a cross-sectional study including a probability sample of adults with VI. The current study had three underlying aims: to assess the prevalence of loneliness in adults with VI; to describe the association of loneliness with sociodemographic factors, VI characteristics, and experiences with bullying and physical or sexual abuse; and to describe the association between loneliness and life satisfaction.

## Methods

### Design and participants

#### Visual impairment population

This cross-sectional study comprised adult members (≥18 years old) of the Norwegian Association of the Blind and Partially Sighted. To achieve full membership, a person needs to present a medical documentation of VI or an irreversible eye condition causing VI [22]. People were excluded if they were deaf, had severe speech impairments, and/or if they did not speak Scandinavian languages or English. Data were collected through structured telephone interviews between January and May, 2017. All interviews were carried out by trained interviewers from a survey company. The majority of members were middle-aged or older [22]. To ensure an adequate number of participants in the youngest age groups, the study participants were divided into different age categories (18–35, 36–50, 51–65, ≥ 66 years), and approximately equal numbers of members across the four age categories were randomly selected within each stratum. Of the 1216 individuals who were contacted, 736 (61%) participated by completing the interview. A flow chart of the sample selection is provided elsewhere [8].

#### General population

We extracted norm data on loneliness and different sociodemographic factors from the Life Course Gender and Generation (LOGG) study [23]. The LOGG study included a national representative sample of non-institutionalized Norwegian adults aged 18–79 years. Data were collected between January 2007 and January 2009 by telephone interviews, self-administered postal questionnaires, and national registries. Of the 25,368 individuals eligible for participation, 14,884 (60.0%) participated in the telephone interview and 10,791 (42.6%) completed and returned the postal questionnaire. We had access to all data from the LOGG study. Data access was granted by the Norwegian Centre for Research Data (Access number: #4392). The LOGG study was approved by the Regional Committee for Medical and Health Research Ethics. Informed consent was obtained from all participants.

### Measurements

#### Loneliness

In the VI population, loneliness was measured using the Three-Item Loneliness Scale (TILS). The TILS was developed for population-based surveys, demonstrating good internal consistency (r = 0.72) and high concurrent validity [24]. The scale consisted of the following three questions: ‘How often do you miss somebody to be with?’, ‘How often do you feel socially excluded?’, ‘How often do you feel socially isolated?’. The response categories were coded 1 (‘hardly ever’), 2 (‘some of the time’), or 3 (‘often’). The scale had a Chronbach’s alpha of 0.81. A sum score was created by summarizing the three items (range 3 to 9). In addition to the continuous loneliness score, we also created a categorical loneliness variable. A score of 5 or 6 was classified as ‘moderate loneliness’ and a score ≥ 7 as ‘severe loneliness’ [25].

The measurements used to assess and classify loneliness in the general population are described in electronic Additional file 1.

#### Life satisfaction

Cantril’s Ladder of Life Satisfaction was employed in the questionnaire to measure current life satisfaction in the VI population [26]. The participants were asked to imagine themselves a ladder with 10 steps, with the bottom of the ladder representing the worst possible life (score = 1) and the top of the ladder representing the best possible life (score = 10).

#### Independent variables

In both the VI population and general population, assessments were made regarding sociodemographic characteristics, including age (18–35, 36–50, 51–65, ≥ 66 years), gender, education (< 11, 11–13, ≥ 14 years), marital status (single, married/partner, divorced/widowed), and occupational status (employed/studying, unemployed, retired).

In the VI population, participants were also asked about the number of household members (1, ≥ 2), past experiences with bullying or physical or sexual abuse (none, bullying only, assaults and bullying), having any other impairments (no, yes), the degree of vision loss (moderate/undetermined, severe, blindness), and the current VI status (stable, progressive). In addition, we created an ‘age at VI onset’ variable by subtracting the participant’s age from their total number of years living with VI. The variable was then categorized into the following three categories: ‘congenital’ (0 years), ‘childhood/youth’ (1 to 24 years), and ‘adulthood’ (≥ 25 years).

### Statistical methods

Descriptive statistics included means, standard deviations (SDs), frequencies and percentages. Pearson’s chi-squared test was used to assess differences in frequency counts. We tabulated the proportion of moderate and severe loneliness within each age category. All proportions were estimated with 95% exact confidence intervals (CIs).

Generalized linear models (GLMs) with Gaussian distribution and a log link were used to estimate independent associations of sociodemographic factors, VI characteristics, and experiences with bullying and physical or sexual abuse with outcomes of loneliness. We treated loneliness as an untransformed continuous variable in the analyses. We evaluated model fit using Akaike’s information criterion and residual plots [27].

We also used Gaussian GLMs to examine the association between loneliness and life satisfaction [27]. These models were either unadjusted or adjusted for age, gender, education, occupational status, marital status, number of people in the household, bullying, physical or sexual abuse, other impairments, VI severity, and years since VI onset. We treated life satisfaction as an untransformed continuous variable in the analyses. A linear dose-response relationship was tested for by comparing the log likelihood between the model with loneliness treated as a continuous variable and the model with loneliness categorized into quartiles and treated as a categorical variable. A *p*-value < 0.05 indicated departure from linearity. We also tested for effect-measure modification between loneliness and each covariate using the likelihood ratio test to compare models with and without the product term [28].

The significance level was set at *p* = 0.05. The statistical analyses were carried out using Stata Version 15 (Stata Corp., Texas, USA).

### Results

Forty-three percent of the VI population had congenital VI and 57% had acquired vision loss during childhood or adulthood. Roughly one in three participants had additional impairments. The age of VI onset ranged from 0 to 76 years (mean 19 years). Thirty-five percent had moderate/other VI, 40% had severe VI, and 25% were blind. Table 1 provides the study characteristics of both the VI population and the general population. The two populations differed significantly in all five study characteristics (*p* < 0.05).

**Table 1:** Study characteristics of the visual impairment population (*N* = 736) and the general population (*N* = 14,884)

	VI population	General population	
Characteristics	n (%)	n (%)	Χ^2^, *p*-value#
Age (years)			86.5, *p* < 0.001
18–35	157 (21.3)	4265 (28.7)	
36–50	186 (25.3)	4568 (30.7)	
51–65	200 (27.2)	3914 (26.3)	
≥ 66	193 (26.2)	2137 (14.4)	
Gender			8.3, *p* = 0.004
Women	333 (45.2)	7545 (50.7)	
Men	403 (54.8)	7339 (49.3)	
Education (years)			33.7, *p* < 0.001
< 11	115 (15.6)	2820 (19.0)	
11–13	286 (38.9)	6849 (46.0)	
≥ 14	335 (45.5)	5215 (35.0)	
Marital status			115.8, *p* < 0.001
Married/partners	347 (47.2)	9580 (64.4)	
Single	260 (35.3)	2953 (19.8)	
Divorced/widowed	129 (17.5)	2351 (15.8)	
Occupational status			522.1, *p* < 0.001
Employed	295 (40.1)	11,061 (74.7)	
Unemployed	271 (36.8)	1633 (7.8)	
Retired	170 (23.1)	2622 (17.7)	

Notes. *VI* visual impairment; #: test statistics and *p*-value derived from Pearson’s chi-squared test

The mean score for loneliness was 4.83 (SD 1.82), 4.88 for women and 4.78 for men (*p* = 0.46). The majority of participants reported missing somebody to be with sometimes (39.0%) or often (21.1%). In addition, high proportions of participants reported being socially excluded sometimes (29.2%) or often (12.2%), and socially isolated sometimes (27.0%) or often (13.3%). As shown in Table 2, the proportion of individuals with VI who were classified as having loneliness was higher than the proportion in the general population. These rates were consistently higher across age groups and severity of loneliness.

**Table 2:** Prevalence of loneliness in the visual impairment population and in the general population

Age categories	VI population (*n* = 736)^a^		General population (*n* = 14,884)^a^			
	TILS		Single question		DCG	
	%	95% CI	%	95% CI	%	95% CI
Moderate loneliness						
18–35 years	31.2	24.1–39.1	19.6	18.4–20.8	16.5	15.3–17.6
36–50 years	30.1	23.6–37.2	15.8	14.8–16.9	16.1	15.1–17.2
51–65 years	23.0	17.4–29.5	17.3	16.1–18.5	18.5	17.3–19.7
≥ 66 years	31.1	24.6–38.1	22.4	20.6–24.2	23.3	21.5–25.1
Total sample	28.7	25.4–32.1	18.2	17.6–18.9	17.9	17.2–18.5
Severe loneliness						
18–35 years	21.0	14.9–28.2	2.1	1.7–2.6	4.9	4.3–5.6
36–50 years	27.9	20.7–33.9	2.2	1.8–2.7	6.1	5.5–6.9
51–65 years	19.0	13.8–25.1	3.2	2.7–3.8	6.0	5.3–6.8
≥ 66 years	12.4	8.1–17.9	3.8	3.0–4.7	7.2	6.1–8.4
Total sample	19.7	16.9–22.8	2.7	2.4–3.0	5.9	5.5–6.3

Notes. *VI* visual impairment, *CI*s confidence intervals, *TILS* Three Item Loneliness Scale, *DCG* De Jong Gierveld Loneliness Scale

^a^The single question had 26 non-responses and the DCG questionnaire had 122 non-responses

Table 3 shows the result of unadjusted and adjusted regression analyses of loneliness across sociodemographic factors, VI characteristics, and past experiences with bullying and physical or sexual abuse. In the unadjusted analyses, all factors, except gender and VI stability, were significantly associated with loneliness. In the adjusted models, those exposed to bullying or those aged 36 to 50 years demonstrated the strongest associations with loneliness. High mean scores for loneliness were also found among those who were blind, had additional impairments, a history of physical or sexual abuse, were unemployed, divorced or widowed, or had 11–13 years of education.

**Table 3:** Regression analyses of factors associated with loneliness in the visual impairment (VI) population (*n* = 736)

	Unadjusted^a^	Adjusted^a^
Independent variables	Beta (95% CI)	Beta (95% CI)
Age (years): 18–35 vs. ≥ 66	**0.44 (0.05, 0.83)**	0.41 (−0.28, 1.11)
36–50 vs. ≥ 66	**0.74 (0.37, 1.11)**	**0.77 (0.12, 1.43)**
51–65 vs. ≥ 66	0.20 (− 0.16, 0.56)	0.23 (−0.40, 0.86)
Gender: Female vs. male	0.10 (− 0.17, 0.37)	− 0.10 (− 0.36, 0.15)
Education (years): < 11 vs. ≥ 14	0.32 (− 0.07, 0.71)	0.16 (− 0.21, 0.53)
11–13 vs. ≥ 14	**0.59 (0.30, 0.88)**	**0.35 (0.07, 0.62)**
Marital status: Single vs. married/partners	**0.89 (0.60, 1.17)**	**0.60 (0.16, 1.03)**
Divorced/widowed vs. Married/partners	**1.01 (0.64, 1.37)**	**1.12 (0.64, 1.59)**
Household members: 1 vs. ≥ 2	**0.66 (0.39, 0.93)**	−0.10(−0.51, 0.31)
Occupational status: Unemployed vs. employed/studying	**0.80 (0.50, 1.10)**	**0.50 (0.20, 0.81)**
Retired vs. employed/studying	−0.07 (− 0.41, 0.28)	0.36 (− 0.30, 1.02)
Other impairments: Yes vs. no	**0.91 (0.64, 1.19)**	**0.66 (0.39, 0.92)**
VI severity: Severe VI vs. moderate VI	0.28 (−0.03, 0.59)	0.16 (−0.13, 0.46)
Blindness vs. moderate VI	**0.37 (0.02, 0.78)**	**0.39 (0.05, 0.73)**
Onset-age VI: Since birth vs. adulthood	0.30 (−0.00, 0.60)	0.09 (−0.22, 0.41)
Childhood/youth vs. adulthood	0.34 (−0.03, 0.72)	0.11 (−0.27, 0.48)
VI stability: Progressive vs. stable	0.13 (−0.17, 0.43)	0.15 (−0.14, 0.43)
Victimized by bullying or abuse: Bullying vs. none	**0.92 (0.62, 1.22)**	**0.76 (0.46, 1.06)**
Assaults vs. none	**1.18 (0.85, 1.52)**	**0.77 (0.43, 1.10)**

Notes. *CIs* confidence interval, *vs* versus

^a^beta values in bold text indicates statistical significance (*p* < 0.05)

Higher scores for loneliness were associated with lower levels of life satisfaction (β = − 0.48, 95% CI: − 0.55, − 0.41). The association was non-linear (*p* = 0.002), with changes in loneliness scores related to greater changes in life satisfaction in the upper part of the loneliness scale (Additional file 1: Figure S2). The estimates remained unchanged after the adjustments (fully adjusted β = − 0.44, 95% CI: − 0.52, − 0.36). In addition, we found a significant product term between loneliness and gender (χ^2^ = 4.2, *p* = 0.04), yielding a slightly stronger association for women (fully adjusted β = − 0.56, 95% CI: − 0.66, − 0.45) than men (fully adjusted β = − 0.36, 95% CI: − 0.45, − 0.21). No other product terms reached statistical significance (*p* > 0.05).

## Discussion

Our findings show that almost one in two adults with VI have moderate or severe loneliness, demonstrating consistently higher rates across age groups than the general population. In addition, the risk of loneliness was higher for those who were aged 36 to 50 years, exposed to bullying or physical or sexual abuse, had blindness, other impairments, or were unemployed. Lastly, high levels of loneliness were associated with lower life satisfaction.

### Interpretation and comparison

The high rates of loneliness in our study are consistent with studies of people with VI in the Netherlands [11, 12], Iceland [16], and Finland [14], and are two to three times higher than rates reported in studies from the US [13] and Canada [18]. Furthermore, the consistently higher rates of loneliness across age groups compared to the general population provide further evidence supporting previous observations that individuals with VI have a higher risk of loneliness compared to sighted individuals [11, 14, 17, 18].

Our findings are in line with the results published by Karlsson [16] showing that people with blindness are more lonely than those with moderate to severe VI. Similar to that study, the majority of participants in our study had a diagnosis of VI since childhood or early adulthood. Studies that included participants who lost their vision late in life have produced mixed evidence with regard to whether VI severity is associated with loneliness [11–13].

The highest level of loneliness being among 36 to 50-year-olds differs from that of the general population, in which loneliness is most common among adolescents and in elderly [29]. Our findings can be interpreted in light of the generally high expectations of being successful in family life, social contexts, and career during this period of life. The feeling of loneliness results from cognitive appraisals in which the individual evaluates the realities against his or her expectations and needs [2]. In older age, vision requirements may not be as crucial. In addition, several participants had lost their sight in their older years, and some of them may have social networks built through a long life. However, we were not able to test these hypotheses empirically.

Higher levels of loneliness among those who were unemployed support the results of a previous report [15] showing that 50% of visually impaired people agreed to the statement that being out of work reduced their social network and resulted in feelings of social exclusion. Lack of enabling environments limited visually impaired people from participating in social life [30], which may be even more demanding with the personal, social, and financial consequences of being excluded from the labour market [31].

In agreement with results from the general population [32], we found exposure to bullying or abuse to be strongly associated with feelings of loneliness. Exposure to negative interpersonal events may induce feelings of social alienation, persistent negative thoughts and emotions, distorted blame of self or others, and loss of trust and faith in oneself, others, or the world [33]. Therefore, difficulties coping with stress reactions could lead to avoidance and withdrawal from social situations, which may result in permanent feelings of loneliness [29].

The strong association between loneliness and reduced life satisfaction is in accordance with documentation from the general population [21], illustrating the importance of social life and connectedness to quality of life. Social interaction is considered to be an integral part of a full-fledged life, and unmet needs could make life less pleasurable and meaningful [34]. However, causality may also be the reverse, in that people who are less satisfied with life could more likely withdraw socially [21].

### Strengths and limitations

The strengths of this study were a large sample size, the probability sampling technique, the use of interview-based methods with validated instruments, and detailed information about several VI-related characteristics. By oversampling younger adults, we were able to obtain valid estimates of loneliness across a broad array of age groups.

This cross-sectional study did not allow us to address relationships of cause and effect, and although we controlled for some potentially confounding factors, it is possible that our analyses were subjected to residual confounding. Furthermore, the use of self-reports on VI and other important factors may have affected the accuracy and validity of the estimates. Although the TIL scale is considered reliable for assessing loneliness [35], it lacks a standardized cut-off for classifying people as ‘lonely’. Our use of a rather conservative cut-off for severe loneliness may have resulted in low estimates for that particular category [25]. Furthermore, non-participation may have introduced bias estimates. Bias of sample selection may primarily affect the frequencies of loneliness and covariates, and to a lesser extent the relationship of interest [36]. Lastly, inclusion of participants from a membership organization of blind and visually impaired people questions the representativeness of our study sample. Compared to 2015 census data from Statistics Norway [37], our study sample did not differ with regard to gender, employment, and geographic location. However, our sample included a higher percentage of people who were blind, highly educated, and living alone.

### Implications

Our findings suggest that coping in social contexts can be more demanding when having difficulties seeing, and that people with VI may be more easily left out and isolated from others. Removing barriers to social participation and integration should be a main objective to prevent social exclusion and loneliness. Such barriers may be general attitudes, legislation, and social, cultural, or physical structures. The concepts of accessibility and universal design are relevant issues in this respect [30]. Moreover, the possible impact of bullying and abuse on loneliness emphasizes a need for preventive measures, as well as professional assistance when such events occur.

The term ‘Information Deprivation Trauma’ is used in the research literature on hard of hearing and deaf individuals and describes how a lack of information in a situation hinders a person from responding appropriately [38]. Consequently, life becomes less predictable or controllable. Information deprivation may also likely affect visually impaired people and contribute to an experience of alienation, exclusion, and loneliness, causal relationships that may mutually reinforce each other. This calls for interventions to improve access to important domains of the environment, including buildings, roads, transportation, and communication [30]. Of particular interest to clinicians is that limited social networking in combination with general information deprivation means that people with VI could experience more problems accessing health care services as well as being less likely to receive optimal health care. Health professionals should be aware of the specific challenges of VI and the importance of good communication and information.

## Conclusions

This cross-sectional study provides new knowledge on loneliness among individuals with VI. Individuals with VI have a higher risk of loneliness across all age groups. We found loneliness to be strongly associated with young age, severe degree of impairment, unemployment, and past exposure to bullying or abuse. Loneliness was also associated with lower levels of life satisfaction. Strategies aiming to promote social participation and accessibility of populations with VI may be beneficial for improving these individuals’ general feeling of well-being.

## Additional file

Additional file 1: Classification of loneliness, relationship between loneliness and life satisfaction, and questions included in the interview guide. (DOCX 117 kb)

## Abbreviations

CI: confidence interval
DCG: De Jong Gierveld Loneliness Scale
LOGG: Life Course Gender and Generation
RR: relative risk
TILS: Three-Item Loneliness Scale
VI: visual impairment
