# Supplementary material for: Loneliness among adults with visual impairment: prevalence, associated factors, and relationship to life satisfaction
Source: Health Qual Life Outcomes. 2019 Feb 1;17:24. doi: 10.1186/s12955-019-1096-y (PMC6359849; doi:10.1186/s12955-019-1096-y)
Supplement: Supplementary file 1 — Classification of loneliness, relationship between loneliness and life satisfaction, and questions included in the interview guide. (DOCX 117 kb) [file 12955_2019_1096_MOESM1_ESM.docx]

**Electronic supplementary material**

***Description and classification of loneliness in the general population***

Data on loneliness from the general population was obtained by using two different measurements; one direct single question and one indirect measure. For the direct single question, the participants were asked to describe whether they felt lonely. The response alternatives were (1) ‘Never’, (2) ‘Rarely’, (3) ‘Sometimes’, and (4) ‘Often’.

For the indirect measure of loneliness, the De Jong Gierveld Loneliness Scale (JGLS) was used. The scale encompasses six negative and positive formulated statements concerning social relationships. The scale had the following five response categories: ‘Strongly agree’, ‘Tend to agree’, ‘Neither agree nor disagree’, ‘Tend to disagree’, and ‘Strongly disagree’. To get a score of 1, the participant had to report on the three last response alternatives on the negative formulated statements (‘neither agree nor disagree’, ‘tend to agree’, and ‘strongly agree’) or to report on the first three response alternatives on the positive formulated statements (‘neither agree nor disagree’, ‘tend to disagree’, and ‘strongly disagree’). Others were given a score of 0. We created a sum score by adding together the six items. The sum score ranged from 0 (not lonely) to 6 (intensely lonely).

As shown in Table S1, those who reported ‘Sometimes’ on the direct question or had a score of 2 or 3 on the JGLS were classified with moderate loneliness, while participants who were classified with severe loneliness reported either ‘Often’ on the direct question or had a score of at least 4 on the JGLS.

**Table S1.** Classification of loneliness across the various measurements.

|  | **Classification of loneliness** | | | |
| --- | --- | --- | --- | --- |
| **Measurements** | **Scale range** | **None** | **Moderate** | **Severe** |
| Three Item Loneliness Scale | 3–9 | 3–4 | 5–6 | ≥ 7 |
| De Jong Gierveld Loneliness Scale | 0–6 | 0–1 | 2–3 | ≥ 4 |
| Direct, single question | 0–3 | Never/rarely | Sometimes | Often |

***The association between loneliness and life satisfaction***

**
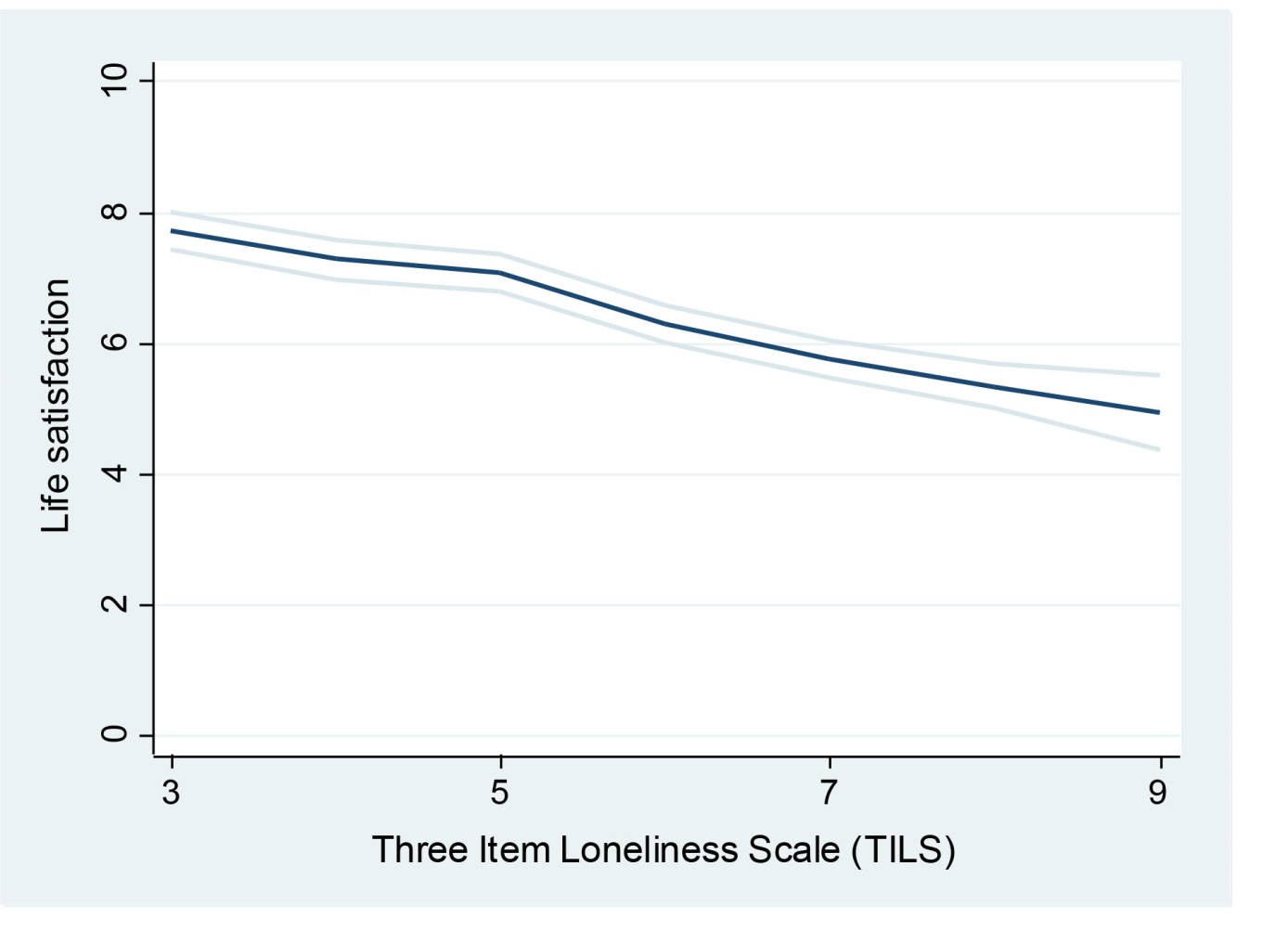
Figure S2.** Running smoothers and corresponding 95% confidence intervals of the association between loneliness and life satisfaction in a population of people with visual impairment (n = 736).

***Type of topics included in the interview***

**Table S2.** Data obtained by the structuralized interview.

| **Topics** | **Data/questionnaires** |
| --- | --- |
| Sociodemographic | 1. Age  2. Gender  3. Marital status  4. Number of people in the household  5. Occupational status  6. Educational attainment  7. Ethnicity |
| Visual impairment | 1. The severity of visual impairment  2. How the visual impairment occurred  3. The cause of the vision loss  4. Years since vision loss  5. The current status of the vision loss  6. Having other functional impairments  7. The use of assistive devices |
| Traumatic events | Life Event Checklist for DSM-5 [1] |
| Post-traumatic stress disorder | PTSD Checklist for DSM-5 [2] |
| Depression | Ten-item Patient Health Questionnaire [3] |
| Bullying | 1. Bullying-items from the General Nordic Questionnaire for psychological and social factors at work [4]  2. Whether bullying was related to the visual impairment |
| Life satisfaction | Cantril’s Ladder of Life Satisfaction [5] |
| General self-efficacy | The General Self-efficacy Scale [6] |
| Loneliness | Three-item Loneliness Scale [7] |
| Social support | The Crisis Support Scale [8] |

**References**

1. Gray MJ, Litz BT, Hsu JL, Lombardo TW. Psychometric properties of the Life Events Checklist. Assessment. 2004;11(4):330-41.

2. Blevins CA, Weathers FW, Davis MT, Witte TK, Domino JL. The Posttraumatic Stress Disorder Checklist for DSM-5 (PCL-5): development and initial psychometric evaluation. J Trauma Stress. 2015;28(6):489-98.

3. Spitzer RL, Kroenke K, Williams JB, Group PHQPCS. Validation and utility of a self-report version of PRIME-MD: the PHQ Primary Care Study. JAMA. 1999;282(18):1737-44.

4. Ørhede E, Hottinen V, Skogstad A, Knardahl S, Elo A-L, Dallner M, et al. User's guide for the QPSNordic: General Nordic Questionnaire for psychological and social factors at work: Nordic Council of Ministers; 2000.

5. Cantril H. A study of aspirations. Sci Am. 1963;208(2):41-45.

6. Schwarzer R, Jerusalem M. Generalized Self-efficacy Scale. In: Weinman J, Wright S, Johnston M (Eds.), Measures in health psychology: a user’s portfolio (p. 35–37). Windsor, UK: NFER-NELSON; 1995.

7. Hughes ME, Waite LJ, Hawkley LC, Cacioppo JT. A short scale for measuring loneliness in large surveys: results from two population-based studies. Res Aging. 2004;26(6):655-72.

8. Joseph S, Andrews B, Williams R, Yule W. Crisis support and psychiatric symptomatology in adult survivors of the Jupiter cruise ship disaster. Br J Clin Psychol. 1992;31(1):63-73.
